# Clinical Trials Targeting Aging

**DOI:** 10.3389/fragi.2022.820215

**Published:** 2022-02-04

**Authors:** Johannes Leth Nielsen, Daniela Bakula, Morten Scheibye-Knudsen

**Affiliations:** Center for Healthy Aging, Department of Cellular and Molecular Medicine, University of Copenhagen, Copenhagen, Denmark

**Keywords:** aging, clinical trials, caloric restriction, NAD, rapamycin, exercise

## Abstract

The risk of morbidity and mortality increases exponentially with age. Chronic inflammation, accumulation of DNA damage, dysfunctional mitochondria, and increased senescent cell load are factors contributing to this. Mechanistic investigations have revealed specific pathways and processes which, proposedly, cause age-related phenotypes such as frailty, reduced physical resilience, and multi-morbidity. Among promising treatments alleviating the consequences of aging are caloric restriction and pharmacologically targeting longevity pathways such as the mechanistic target of rapamycin (mTOR), sirtuins, and anti-apoptotic pathways in senescent cells. Regulation of these pathways and processes has revealed significant health- and lifespan extending results in animal models. Nevertheless, it remains unclear if similar results translate to humans. A requirement of translation are the development of age- and morbidity associated biomarkers as longitudinal trials are difficult and not feasible, practical, nor ethical when human life span is the endpoint. Current biomarkers and the results of anti-aging intervention studies in humans will be covered within this paper. The future of clinical trials targeting aging may be phase 2 and 3 studies with larger populations if safety and tolerability of investigated medication continues not to be a hurdle for further investigations.

## Introduction

As age increases, so does the susceptibility to a series of chronic diseases which ultimately result in fatal endings. This is such a basic part of life that we rarely consider if there is anything we can do to postpone it. So far, the principal of “one-disease-one-treatment” has brought medical sciences far but this line of thought may soon be outdated when it comes to aging related conditions. It is like fighting a many-headed monster: If one condition is treated successfully, another emerges shortly after. This point is illustrated as eradicating the two leading causes of death (cancer and cardiovascular disease) extends mean life span by 3.3 and 4 years, respectively (Arias et al., 2013). Interestingly, the gain of treating multiple diseases combined exceeds the sum of these numbers.

Aging is the greatest risk factor for most diseases likely because as aging progresses, cells and tissue undergo a series of processes which result in gradually declining functionality, accumulation of damage, increased inflammation, and cell death. If these processes are reversable or treatable, all aging related chronical diseases may potentially be simultaneously treated—or postponed—and healthy aging could be achieved. This approach to treating aging itself could effectively treat chronic diseases among the world’s elderly, shifting from treating symptoms of aging to treating the cause of it. The fact that the number and proportion of elderly people (>65 years) is growing in every country in the world underlines the relevance of this field of research (World Population Prospects - Population Division, 2021).

**FIGURE 1:**
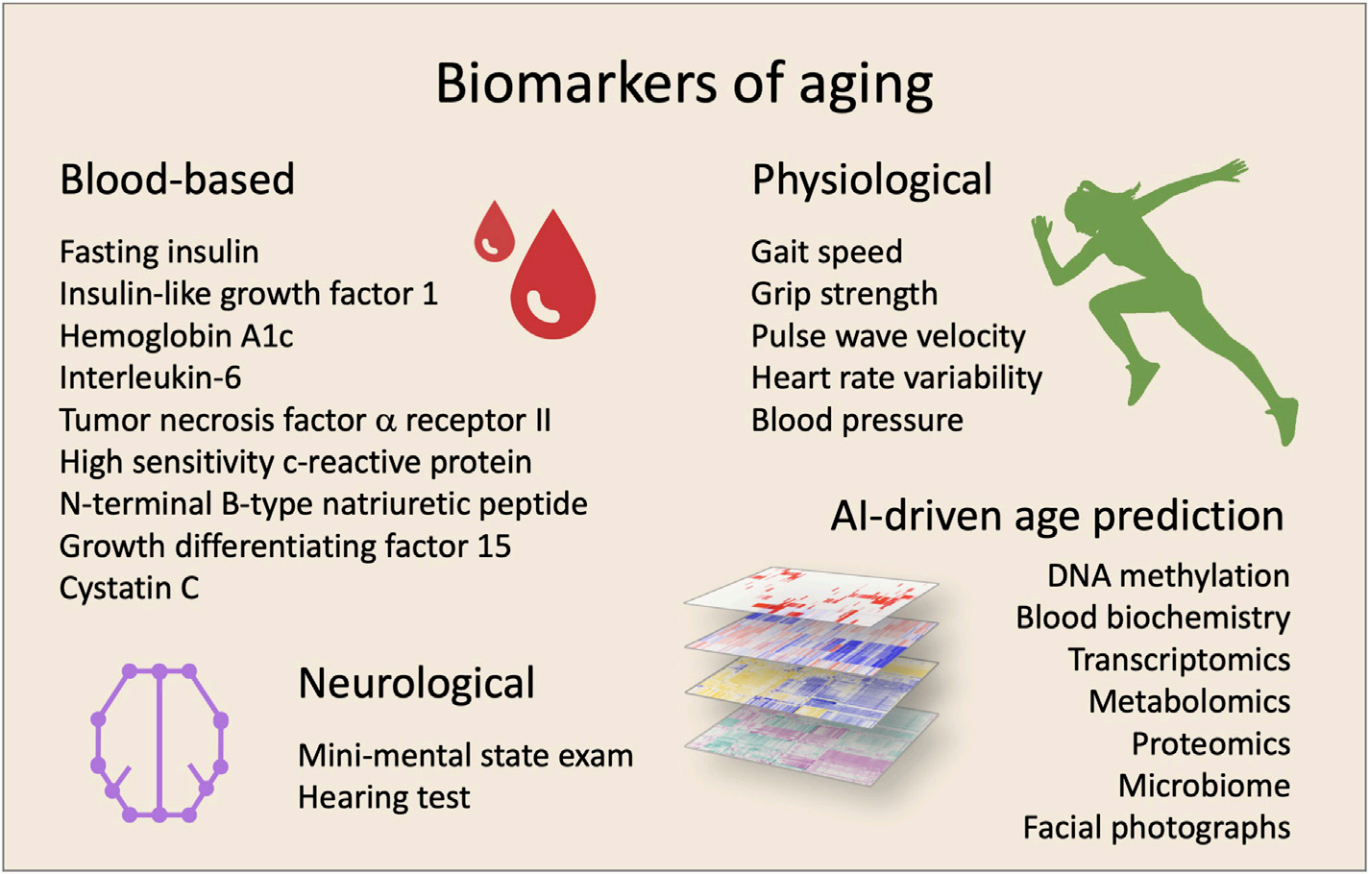
Biomarkers for clinical trials targeting aging. The effectiveness of interventions can be evaluated by using a combination of biomarkers. In the last years, different biomarkers have been proposed using various sample- and measurement-types.

If it is possible to treat the underlying causes of aging it is likely to achieve a prolonged health- and lifespan. Yet, practical and ethical issues are major bumps on the road to longevity as trials in humans are being initiated. It is not feasible for clinical studies to have lifespan as a primary outcome. Surrogate biomarkers of lifespan—or more importantly, health span—are necessary for human trials to advance. Recently, a selection of biomarker candidates for monitoring aging treatment efficacy were suggested including blood biochemistry markers for inflammation, such as c-reactive protein (CRP), and metabolism, such as insulin like growth factor-1 (IGF-1) (Justice et al., 2018). Such biomarkers need to be correlated with aging and morbidity, easily accessible *in vivo*, respond to treatment, and present parallel changes with susceptibility to disease. In addition to single biomarkers, computational power and machine learning algorithms have allowed the development of highly accurate complex biomarkers of aging. These include whole-genome DNA methylation (Horvath and Raj, 2018) and transcriptomic changes as well as other omics-based analyses (Osborne et al., 2020). Further, simple facial photographs appear to accurately predict not only age but also morbidity (Dykiert et al., 2012). In addition, machine learning analyses of a panel of standard blood biochemistry markers also appear to be able to predict age and morbidity (Mamoshina et al., 2018). Excitingly, after decades of work it now appears that we have the possibility of measuring biological age (Figure 1).

While good biomarkers are just now being described, clinical trials have already been completed that attempt to target aging. In the following we will describe key trials that have attempted to target aging (Table 1). We will specifically focus on interventions where there is strong preclinical evidence for an effect on aging. However, we encourage the reader to also investigate emerging interventions such as stem cell reprogramming (Sarkar et al., 2020), senescence immunotherapy (Song et al., 2020), microbiome augmentation (Wilmanski et al., 2021) as well as more targeted nutraceutical interventions (Sharma and Padwad, 2020) that could prove revolutionary for healthy aging.

**TABLE 1:** List of clinical trials targeting aging.

Intervention	Outcome	References
CR	Glucose ↓, Blood pressure ↓	Wierik et al. (1994)
CR	Glucose ↓, Blood pressure ↓, resting metabolic rate ↓	Loft et al. (1995)
CR	Insulin ↓	Racette et al. (2006)
CR	T_3_ ↓, T_4_ ↓, Body temperature ↓, Mitochondria ↑, resting metabolism ↓	Civitarese et al. (2007)
CR	Cholesterol ↓, Blood pressure ↓	Lefevre et al. (2009)
CR	Insulin ↓, body temperature ↓, resting metabolic rate ↓	Heilbronn et al. (2006)
CR	DNA methylation pace of aging ↓	Waziry et al. (2021)
CR	Body mass ↓, IGF-1/IGFBP1 ↓, IGFBP1 ↑, cortisol ↓	Fontana et al. (2016)
CR	Body mass ↓, cholesterol ↓, blood pressure ↓, CRP ↓, insulin sensitivity ↑	Waziry et al. (2021)
NR + PT	NAD ↑, liver enzymes ↓, blood pressure↓	Dellinger et al. (2017)
NR + PT	No effect on muscle regeneration	Jensen et al. (2021)
NR	NAD ↑	Martens et al. (2018)
NR	NAD (blood) ↑, NAD (muscle) -, IL-2 ↓, IL-5 ↓, IL-6 ↓,TNF-α ↓	Elhassan et al. (2019)
NR	No effect	Dollerup et al. (2018), Dollerup et al. (2020)
NR	Mitochondria ↑, IL-1B ↓, IL-6 ↓, IL-18 ↓	Zhou et al. (2020)
D + Q	Gait speed ↑, Walking distance ↑, Char stand ↑	Justice et al. (2019)
D + Q	p16 ↓, p21 ↓, IL-1α, IL−2 ↓, IL−6 ↓, IL-9 ↓, MMP-2 ↓, MMP−9 ↓, MMP−12 ↓	Hickson et al. (2019)
RAD001 + BEZ235 (mTOR inhibition)	Infections ↓, Immune response ↑	Mannick et al. (2018)
Rapamycin (topical)	p16 ↓, Collagen ↑	Chung et al. (2019)
UA	Acylcarnitine ↓, mitochondria ↑	Andreux et al. (2019)
Exercise	IL-8 ↓	Balan et al. (2020)
Exercise + diet	DNA methylation age ↓	Fiorito et al. (2021)
Exercise + diet + sleep + phytochemicals	DNA methylation age ↓	Fitzgerald et al. (2021)

## Calorie Restriction

Calorie restriction (CR) is a dietary intervention with sufficient nutrition but reduced overall calorie content that has been shown to extend the lifespan of organisms from yeast to primates (de Cabo and Mattson, 2019). Throughout the 1990s, a series of CR studies investigated the effect of various grades of CR on glucose metabolism, blood pressure, and other vital parameters in humans. Reductions in glucose concentrations, systolic and diastolic blood pressure were observed (Wierik et al., 1994; Loft et al., 1995). Both body mass and resting metabolic rate were reduced. Interestingly, the resting metabolic rate decrease was greater than expected when adjusting for weight (residual resting metabolic rate), indicating possible CR-induced metabolic adaptive mechanisms. The Washington University CALERIE group conducted a 12-month randomized trial with 48 healthy, overweight, non-obese (mean BMI 27.3 kg/m^2^), middle-aged individuals (Racette et al., 2006). Accordingly, a decrease in fasting insulin and improved insulin sensitivity has been reported after CR. However, TNF-α levels showed no changes between the CR and control group, suggesting no effect of CR on inflammation (Weiss et al., 2006). CR also caused reductions in plasma T_3_ and T_4_ levels at 3 and 6 months, and core body temperature and DNA damage were significantly reduced among all intervention groups. Decreased activity of the antioxidant superoxide dismutase (SOD) suggested reduced oxidative stress (Civitarese et al., 2007), although protein carbonylation (another marker of oxidative stress) remained unchanged (Heilbronn et al., 2006). Gene expression of proteins involved in mitochondrial functioning, (SIRT1, endothelial nitric oxide synthase, and Presenilin-associated rhomboid-like protein, PARL) increased after CR, along with mtDNA levels which increased by 35%. Though, no changes were observed in the activity of key enzymes involved in the Krebs’ cycle (citrate synthase), beta oxidation (beta-hydroxyacyl-CoA dehydrogenase), or electron transport chain (cytochrome C oxidase II). In general, it appears that CR confers diverse health benefits upon metabolism and inflammation.

CR caused a change in lipid profile, reducing low density lipoprotein (LDL) cholesterol and triglyceride levels and increasing high density lipoprotein (HDL) cholesterol levels (Lefevre et al., 2009; Kraus et al., 2019). This was also observed by Kraus et al. with cardiovascular markers improved with reduced diastolic blood pressure and a decrease in CRP (Kraus et al., 2019). However, one study found that inflammatory markers (hsCRP, TNF-α, IL-6) increased with only IL-6 changes reaching significance (Larson-Meyer et al., 2008). Furthermore, adiponectin levels were increased significantly and despite its increasing effect on energy expenditure, residual resting metabolic rate was reduced in all intervention groups and not in the control group (Civitarese et al., 2007). A decrease of resting metabolic rate was observed by Heilbronn et al., but CR led to a decrease 6% greater than expected among intervention groups compared to control groups, indicating metabolic adaptions, similar to what Loft and colleagues observed 2 decades earlier (Loft et al., 1995; Heilbronn et al., 2006). Work from rhesus monkeys have suggested that glycemic load might impact lifespan extension in response to CR (Mattison et al., 2017). However, in a 6 months trial on humans exposed to high glycemic load CR and low glycemic load CR both interventions led to similar changes in markers of oxidative stress (Meydani et al., 2011).

Calorie reduction induces metabolic, inflammatory changes proposed to extend life span in animal models (de Cabo and Mattson, 2019). Randomized, placebo-controlled, clinical trials in humans have produced similar results, as cardiovascular factors have improved (blood pressure and lipid profile) and metabolism has been more efficient (increased insulin sensitivity). More recently, a trial investigated the effect of CR on DNA methylation markers of aging was performed on a considerable group of individuals (*n* = 175). Somewhat disappointingly, only changes in some DNA methylation biomarkers were observed perhaps suggesting that CR mainly affects healthspan and not the pace of aging (Waziry et al., 2021). In total, biomarkers suggest promising health-improving results of CR in humans, however, the feasibility of CR in humans outside of a clinical setting is quite limited. Thus, CR mimetics, such as metformin are of huge interest. Metformin is an established anti-diabetic drug which has improved health and lifespan substantially in mice (Martin-Montalvo et al., 2013). Interestingly, a study has shown that human diabetics treated with metformin live longer than non-diabetics not treated with metformin, suggesting that metformin not only treats diabetes but provides health-gaining effects beyond that (Bannister et al., 2014). A multi-center, 6-year clinical trial with 3,000 subjects [Targeting Aging with Metformin (TAME)] is soon to be underway and will specifically investigate the anti-aging effects further (Justice et al., 2018). Further, altering dietary patterns with intermittent fasting periods has shown considerable potential as an intervention that is easier to implement than CR but yields the same benefits (de Cabo and Mattson, 2019). It will be exciting to see how these interventions affects human aging in larger cohorts.

In sum, CR appears to increase parameters of health in humans yet the effect of CR on biomarkers of aging are still under investigation.

## NAD^+^ Supplements

NAD^+^ and NADH are the oxidized and reduced forms, respectively, of the metabolite nicotinamide adenosine dinucleotide (NAD). NAD^+^ is an important indicator of intracellular energy. NAD^+^ levels deplete with age perhaps due to age-associated mitochondrial dysfunction (Zhu et al., 2015), increased expression of the NAD metabolizing enzyme CD38 (Covarrubias et al., 2020) or through an accumulation of DNA damage driving NAD^+^ depletion *via* PARP1 activation (Fang et al., 2014; Scheibye-Knudsen et al., 2014; Keijzers et al., 2017). Importantly, NAD levels can be increased through ingestion of biochemicial precursors nicotinamide riboside (NR) and nicotinamide mononucleotide (NMN) (Petr et al., 2020). Notably, increasing NAD levels has been shown to extend the lifespan of mice (Zhang et al., 2016) and improve physological parameters in old animals (Gomes et al., 2013).

The safety and efficacy of the combination of 250/500 mg NR and 50/100 mg pterostilbene (PT, a polyphenol in blueberries NRPT) was investigated among 120 elderly subjects between the ages of 60 and 80 years (Dellinger et al., 2017). The eight-week, randomized, double-blinded, and placebo-controlled study showed that in general the intervention was safe. NRPT treatment elevated NAD^+^ levels in whole blood in a dose dependent manner and no change in NAD^+^ levels was observed in the placebo group. Interestingly, low dose NRPT showed a significant reduction in circulating levels of the liver enzyme alanine transaminase (ALAT) and a significant reduction in diastolic blood pressure at day 60. Higher dose NRPT improved mobility of the elderly individuals. Obviously, it is unclear if these interesting effects stem from PT or NR treatments.

NRPT was also tested in the context of muscle injury in the elderly. Thirty-two elderly men and women were recruited to the study and treated with 1,000 mg/200 mg of NRPT (Jensen et al., 2021). After 2 weeks of treatment muscle injury was induced through electric stimulation and muscle biopsies were collected at multiple time intervals after the injury. While the injury was clearly observable no effect was seen of the NRPT treatment.

Another double-blinded trial on 30 subjects investigated if daily 1 g NR supplementation for 6 weeks is tolerated and stimulates NAD^+^ metabolism in healthy, normal weight, middle-aged and older humans (Martens et al., 2018). No adverse effects were reported, and no differences were seen between NR or placebo treatment in hematology, kidney function, liver enzymes, and lipid profile, and clinical laboratory values never exceeded the normal reference intervals. Isolation of peripheral blood mononuclear cells revealed a significant 60% increase in NAD^+^ levels in the NR group compared to the placebo group. Martens and his colleagues observed no effects of NR treatment on markers of metabolic function, exercise capacity, BMI and fat percentage or overall motor function compared to placebo.

Another, smaller study suggested similar conclusions. Twelve overweight, elderly, healthy men were given 1 g NR supplement for 21 days in a placebo-controlled, double-blinded, randomized, crossover study with a 21-day washout period between NR and placebo treatment (Elhassan et al., 2019). NR was well tolerated during the treatment with no adverse effects reported. Clinical biochemical analysis revealed no changes of hematology and kidney, liver, and thyroid function. Whole venous blood, urine, and skeletal muscle were analyzed for NAD^+^ levels. In whole blood, NAD^+^ levels doubled, nicotinic acid adenine dinucleotide (NAAD) levels increased 4.5-fold, and NMN increased 40% during NR treatment. Notably, in skeletal muscle, NR supplement did not increase NAD^+^ levels compared to placebo, but concentrations of NAAD doubled.

Cell motility, cell adhesion, and actin cytoskeleton organization genes were upregulated upon NR treatment in skeletal muscle. Genes related to energy metabolism (glycolysis, Krebs’ cycle, mitochondria) were significantly downregulated and no change in transcription of key genes of NAD^+^ metabolism was found. It has previously been reported that glycolysis increases in mouse cardiac cells upon NR treatment (Diguet et al., 2018), but the results of Elhassan et al. did not support this, as the expression of glycolytic enzymes was unchanged. Skeletal muscle biopsies revealed no change in mitochondrial bioenergetics after NR treatment. Nor was sirtuins (NAD^+^-dependent protein deacetylases) activity changed in skeletal muscle in the NR group. Some proinflammatory biomarkers saw reduced levels, as IL-6, IL-5, IL-2, and TNF-α decreased significantly upon NR treatment compared to baseline values. Other biomarkers of inflammation, including hsCRP, remained unchanged. Fasting glucose, fasting insulin, insulin resistance, blood pressure, body weight, lipid profile remained unchanged. Physical markers of frailty did not change due to NR either, as hand grip and body-weight-adjusted relative strength did not differ between groups (Elhassan et al., 2019). Though, this is not surprising as physical strength is not expected to increase after 21 days of supplement and no muscle training, supported by a meta-study on sarcopenia, suggesting a minimum three-month intervention in order to improve muscle strength (Yoshimura et al., 2017). Interestingly, in a study of heart failure patients NR also appeared to decrease markers of inflammation although the study is quite underpowered (Zhou et al., 2020).

NAD^+^ depletion is suspected as a factor of aging-related diseases, causing metabolic dysfunction. The effects of NR supplement on insulin sensitivity and other metabolic parameters were investigated in a 12-week study (Dollerup et al., 2018). NR supplement (2 g/day) or placebo was given to 40 healthy, sedentary, obese, insulin-resistant men. NR did not change insulin sensitivity, rate of lipolysis, resting energy expenditure, respiratory exchange ratio, HbA1c, fasting glucose, insulin, ALAT, body composition or cholesterol levels compared to placebo. In a follow up study, Dollerup et al. further investigated the effect of NR on mitochondrial function in obese and insulin resistant men and observed no effect of 1 g NR per day for 12 weeks (Dollerup et al., 2020).

NR is not the only NAD metabolite well-tolerated in humans. Ten healthy men were monitored for 5 h after non-blinded oral consumption of either 100, 250, or 500 mg nicotinamide mononucleotide (NMN). The doses were well tolerated and did not result in severe AEs (Irie et al., 2020).

To summarize progress on human trials with NAD supplement, researchers are still at the early stage of human studies, investigating the safety and dosage of NAD^+^-increasing molecules. So far, NR supplementation, NAD^+^ levels and metabolites related hereto have been elevated significantly as a result of oral NAD supplement. Effects of increased NAD^+^ levels on metabolic and mitophagic pathways through exogenous supplements have not been demonstrated and remain to be investigated thoroughly in large-scale trials, covering longer periods of time to investigate potential benefits beyond a limited treatment duration.

## Targeting Senescent Cells With Senolytics

An accumulation of senescent cells is a hallmark of aging. They can occur due to damage-associated signals like DNA damage, telomere erosion, ROS accumulation, NAD^+^ depletion, inflammatory signals, pathogen-associated molecular patterns, damage-associated molecular patterns, oncogenes, and signals from dysfunctional mitochondria (McHugh and Gil, 2018). Importantly, removal of these cells using genetic or pharmacological (so called senolytics) interventions appear to extend the health- and lifespan of mice and these approaches have recently begun to be translated to humans.

Idiopathic pulmonary fibrosis is a fatal disease associated with aging related hallmarks including telomere attrition, oxidative stress, DNA damage, inflammation, and particularly cellular senescence (Waters et al., 2018). Studies of idiopathic pulmonary fibrosis mice indicate positive effects of the senolytic drugs dasatinib and quercetin (D + Q) on pulmonary and physical function (Schafer et al., 2017) and a study with idiopathic pulmonary fibrosis patients, mainly investigating the safety of D + Q treatment, suggests beneficial effects on physical function in humans as well (Justice et al., 2019). Fourteen idiopathic pulmonary fibrosis patients were treated with D + Q for 3 weeks and the results showed not only significant but also clinically meaningful improvements in physical function including 6-min walking distance, 4-m gait speed, and chair-stand time. The study showed no effect of D + Q treatment on pulmonary function, clinical chemistries, reported health, and frailty. The study was not initially intended to investigate effects on circulating senescent associated secretory phenotype (SASP, pro-inflammatory markers of cellular senescence) factors but weak indications of such were suggested (Justice et al., 2019).

Increased senescent cell burden is associated with diabetes and kidney dysfunction, especially in adipose tissue of these patients. A clinical trial investigated the effect of D + Q on adipose tissue senescent cells in humans (Hickson et al., 2019). Eleven subjects with diabetes and kidney dysfunction were treated with D + Q for 3 days. Results revealed a significant decrease in markers of senescence and further analysis of the cellular composition supported a significantly decreased senescent cell burden in adipose tissue. Finally, the study supported a suggestion proposed by Justice et al. (2019), as D + Q significantly reduced circulating SASP factors in patients.

Testing of senolytics in humans have so far shown promising results in improving physical function in idiopathic pulmonary fibrosis patients and reducing senescence load in patients with diabetes and kidney dysfunction. Non-pharmaceutical interventions have also been proposed as senescence-reducing treatments: Endurance training did seem to affect inflammation in skeletal muscle, but no changes on senescence was observed. In a recent study, preliminary results indicate the failure of a phase two trial investigating 12-week senolytical treatment of knee osteoarthritis, with the full results yet to be presented (UNITY, 2021) Biotechnology Announces 12-week data from UBX0101 Phase 2 Clinical Study in Patients with Painful Osteoarthritis of the Knee Unity Biotechnology. There could be many reasons why the trial failed, perhaps the approach is simply not feasible for this disease, perhaps the drug concentrations used were wrong, perhaps the inclusion criteria could have been tighter or wider etc., With that being said, clinical trials with senescence-reducing interventions are still at an early stage and need further investigation. Though, drug tolerance and safety have not been issues, paving the way for future trials.

## Clinical Trials Targeting mTOR

mTOR inhibition with rapamycin has shown promising life extending results in animal models (Harrison et al., 2009). Rapamycin has been used in the clinic as an immunosuppressant for kidney transplant and the side effect profile is relatively well studied in this patient population (Li et al., 2014). However, mTOR inhibitors such as rapamycin has also been tested in healthy elderly with little to no side effects reported after up to 8)weeks of treatment (Kraig et al., 2018; Mannick et al., 2018). A main concern inhibiting mTOR in the elderly is the possibility of immune suppression. However, six-week treatment with an mTOR inhibitor revealed promising results in 264 elderly individuals (Mannick et al., 2018). Whole blood mRNA sequencing analysis showed that genes involved in anti-viral immune response were upregulated significantly in the treatment group, and infections rates were reduced significantly compared to placebo treatment. No significant effects on inflammatory markers were observed as IL-6, interferon-gamma, TNF-α, and IL-18 concentrations remained unchanged. Furthermore, the immune response to an influenza vaccine showed significant improvements in the treatment group as hemagglutination inhibition tests showed increased anti-viral antibody response. The results indicate that TORC1 inhibition improves systemic immune function in the elderly with decreased infection rates and upregulated immune function perhaps due to an augmentation of interferon mediated viral defences (Mannick et al., 2018).

Further clinical evidence for mTOR as a target for aging comes from topical rapamycin treatment on senescence in aging human skin (Chung et al., 2019). Seventeen subjects over 40 years and with age-related photoaging of skin applied rapamycin-containing hand cream to the dorsal side of the same hand and placebo hand cream to the other, daily or every other day for 8 months. The rapamycin-treated hands showed significantly reduced p16^INK4A^ protein levels, indicating reduced skin senescence. Collagen VII protein, critical to the basement membrane integrity, increased significantly upon rapamycin treatment, which was supported by clinically improved skin appearances as the treated hands appeared younger than the other on photographs. Immunohistochemical analysis revealed histological improvements of skin tissue. The results indicated that rapamycin treatment potentially has anti-aging effects in humans—at least in skin.

A main effect of mTOR inhibition is the activation of autophagic pathways such as mitophagy that removes damaged mitochondria. Functional mitochondria are essential to a healthy organism as they are important in oxidative metabolism, lipid metabolism, production and handling of ROS, and apoptotic signaling pathways (Wallace et al., 2010). With age, the efficiency of mitophagy mechanisms decrease and faulty mitochondria accumulate. This aging-related process is closely associated with slowed walking speed, reduced muscle strength, and other aging phenotypes in humans (Coen et al., 2013; Kauppila et al., 2017; Zane et al., 2017; Bakula and Scheibye-Knudsen, 2020). The AMPK-SIRT1 axis is involved in mitochondrial health as activation hereof induces mitophagy. AMPK induces the PGC-1α transcription factor, a regulator of autophagy, while SIRT1 activates UCP-2 (uncoupling protein 2), stimulates mitophagy, and alleviates aging features in preclinical models (Fang et al., 2014; Scheibye-Knudsen et al., 2014). Currently, the strategies of treating declining mitophagy include NAD^+^ supplements, activation of AMPK and/or SIRT1 via other substrates, and mTOR inhibition. Urolithin A (UA) proposedly inhibits mTOR and has shown promising improving results on mitochondrial health, life span, and endurance capacity in animal models (Ryu et al., 2016) and is generally recognized as safe by the US Food and Drug Administration. A 2019 clinical trial showed significant improvements of UA treatment on mitochondrial health as plasma acylcarnitine (an inverse indicator of mitochondrial function and fatty acid oxidation capacity) levels decreased and skeletal muscle expression of essential mitochondrial genes increased, supported by microarray analysis of mRNA levels (Andreux et al., 2019). Quantitative PCR (qPCR) analysis showed that mtDNA levels increased significantly. Interestingly, similar trends have been observed in physically active humans as endurance training triggers mitochondrial biogenesis and improved fatty acid oxidation (Jeppesen et al., 2012).

## Exercise

Endurance training is associated with reduced systemic and muscle inflammation, and telomere-improving mechanisms can be seen in long-term high-intensity cross country skiers (Beavers et al., 2010; Østhus et al., 2012). A recent study investigated if endurance training affects senescent cells in skeletal muscle (Balan et al., 2020). Thirtyfour men were divided into four groups based on age and cycling experience (young sedentary, old sedentary, young trained cyclist, and old trained cyclist) and skeletal muscle biopsies were taken. Quantification of telomere-associated DNA damage and SA-β-Gal staining showed increased senescent cell levels in older individuals, unaffected by endurance training status. Similarly, mRNA levels of *p16* and *p21* increased with age (4-fold and 2-fold, respectively), independent of training status, suggesting increased senescence. Old age was also associated with doubled *CD68* mRNA levels, hinting increased macrophages in skeletal muscle, possibly due to increases in SASP. With regards to inflammatory status, training did seem to play a role as *IL-8* mRNA levels were significantly reduced by 70% in trained individuals compared to sedentary. *TN*F-α mRNA levels were decreased by 40% in trained individuals, but the change did not reach significance. Overall, the study argues that endurance training reduces inflammation in young and old men while senescence levels increase with age and are perhaps unaffected by endurance training.

Recently, reversal of DNA methylation age has been shown in a randomized trial on 43 adult males aged 50–72 subjected to multiple interventions targeting diet, sleep, exercise and others (Fitzgerald et al., 2021). Here, the intervention cohort showed a 3.23 year age reversal. These findings have been corroborated in a larger 2-year study on 219 women subjected to diet and exercise where the intervention group showed significant reduction in a marker of DNA methylation age (Fiorito et al., 2021). Clearly, these prospective trials show that epigenetic markers of aging can indeed be reversed.

## Discussion

Clearly, several trials have shown that targeting aging is feasible in humans. Calorie restriction has been associated with protective cardiovascular effects (lowered blood pressure and improved lipid profile), improved mitochondrial biogenesis and metabolic efficiency (increased insulin sensitivity). A drawback of calorie restriction is that the feasibility is quite low for most humans. Of note, some evidence suggests that the Mediterranean diet, a diet rich in greens, fish, nuts and oil, may affect similar processes as caloric restriction with a reduction in cardiovascular risk (Estruch et al., 2018). Recently, an improvement of the immune function and an augmented gut microbiome has been suggested as possible mechanisms underlying the beneficial effect of the Mediterranean (Maijo et al., 2018; Ghosh et al., 2020). However, altering dietary patterns can be challenging and calorie restriction mimetics may be a promising alternative. NAD^+^ supplements are safe in humans and increase NAD^+^-related metabolites but the influence on cellular energy-sensing pathways, and aging itself, has not shown clear results. Trials with senolytics have shown promising systemic results in subjects with idiopathic pulmonary fibrosis, diabetes, and kidney dysfunction. Nevertheless, senescence is an essential anti-cancer mechanism and interfering with this may be associated with cancer development (Heckenbach et al., 2021). Also, senolytics might even cause further inflammation in already inflamed tissues if a compromised immune system (due to immunosenescence) may not be able to handle increased loads of apoptotic bodies following senolytica-induced apoptosis of senescent cells (McHugh and Gil, 2018). Other potential side effects are delayed skin healing and unwanted targeting of proliferating cells (Zhu et al., 2017). Although senolytics are actively developed for anti-aging treatment, clinical researchers must step carefully in future regulation of these pathways. Further, mTOR inhibition causes improved mitochondrial function, dermatological skin improvements and overall improved immune function in elderly individuals, possibly by lowering immunosenescence.

An important feature of potential aging drugs must be a relative absence of side-effects. Here, the benefit of utilizing wide-spread drugs that are approved for treatment of other diseases is that safety and tolerability is already thoroughly investigated, making it possible to commence larger-scale trials sooner. However, if treatment duration is prolonged periods of time, the health gaining effects must outweigh potential side-effects. For example: Dasatinib can cause gastrointestinal bleeding and liver damage (Liu et al., 2020). A possible approach to avoid this may be combining medication, i.e., handling rapamycin-caused glucose dysmetabolism with metformin (Blagosklonny, 2019).

A bump on the road for expansion of aging trials is the inclusion of mainly healthy subjects in current anti-aging clinical trials, as long lists of wide-spread morbidities and medication often are among exclusion criteria. Thus, studies may include only exceptionally healthy elderly where effects of therapies targeting aging may be less efficient. Further, this could cause a blind-spot in catching potential side-effects of aging treatments as the side-effects may be related to other conditions (Vaiserman et al., 2021). Instead, one could consider having slightly less stringent inclusion criteria which would allow individuals with mild chronic diseases (eg. hypertension) to be included. Similarly, an estimated 1/3 of all elderly receive five or more prescription drugs, potentially resulting in missed drug-drug interactions (Maher et al., 2014).

Medication often has a therapeutical concentration window, where too little poses no effect and too much is toxic (Gagne et al., 2013). The same principle is relevant in anti-aging treatment but may include an additional temporal aspect. Initiation of some anti-aging treatments may require early intervention and might not be efficient if subjects are already old, while other treatment forms may show promising results in the elderly but cause unwanted, harmful side-effects in healthy, young subjects. This temporal therapeutical window has been experimentally observed in cancers (Lin et al., 2020) and could cause major issues for anti-aging clinical trials. If no effect of a treatment is observed due to “incorrect” age of subjects or if it simply has no effect in any age-group can only be investigated by comparing identical studies on different age-groups. Preclinical studies might assist in hinting at potential age-windows for treatment, but clinical trials have to face the issue in some degree (Vaiserman et al., 2021).

In conclusion, clinical trials targeting aging in humans have shown promising but limited results on biomarkers so far. With the emergence of AI driven complex age- and health-predictors that finally allows us to accurately measure the aging process, this will likely change. Nevertheless, biomarkers should be chosen carefully considering the specific trial that is investigated and the likelihood of affecting that specific marker. Indeed, as pointed out a list of biomarkers for monitoring healthy aging has been proposed and shown to be mutable in patients population, suggesting that aging can indeed be targeted in humans.
